# Characterization of Carbapenem-Resistant* Acinetobacter baumannii* Strains Isolated from Hospitalized Patients in Palestine

**DOI:** 10.1155/2017/8012104

**Published:** 2017-07-26

**Authors:** Regeen Handal, Lulu Qunibi, Ibrahim Sahouri, Maha Juhari, Rula Dawodi, Hiyam Marzouqa, Musa Hindiyeh

**Affiliations:** ^1^Caritas Baby Hospital, Bethlehem, State of Palestine; ^2^Bethlehem University, Bethlehem, State of Palestine; ^3^Alia Hospital, Hebron, State of Palestine; ^4^Hussein Hospital, Bethlehem, State of Palestine; ^5^Rafidia Hospital, Nablus, State of Palestine; ^6^Palestinian Forum for Medical Research, Ramallah, State of Palestine

## Abstract

The American Centers for Disease Control and Prevention (CDC) recognizes* Acinetobacter baumannii* as a source of global outbreaks and epidemics especially due to its increasing resistance to commercially available antibiotics. In this study, 69 single patient multidrug resistant isolates collected from all over Palestine, except Gaza, were studied. All the isolates were resistant to all the *β*–lactam antibiotics including the carbapenems. Of the 69 isolates, 82.6% were positive for *bla*_OXA-23_, 14.5% were positive for *bla*_OXA-24_, and 3% were positive for *bla*_OXA-58_. None were positive for *bla*_OXA-143_ and *bla*_OXA-235_. In addition, 5.8% and 0% were positive for *bla*_NDM_ and *bla*_KPC_, respectively. Of the 69 isolates, none were positive for the aminoglycoside* aphA6* gene while 93% were positive for the* aphA1* gene. The acetyltransferases* aacC1* and* aacA4* genes tested positive in 22% and 13% of the isolates, respectively. The* ompA* biofilm-producing virulence gene was detected in all isolates. Finally, Multilocus Sequence Typing (MLST) of 13 isolates revealed that more than one strain of* A. baumannii *was circulating in Palestinian hospitals as results revealed that 7 isolates were of ST208, 2 isolates ST218, 1 isolate ST231, 1 isolate ST348, and 2 new Sequence Types. The detection of these drug resistant pathogens is a reminder of the importance of active surveillance for resistant bacteria in order to prevent their spread in hospital settings.

## 1. Introduction

*Acinetobacter baumannii* is a gram-negative, nonfermentative, oxidase-negative coccobacillus. The Centers for Disease Control and Prevention (CDC) recognizes multidrug resistant (MDR)* A. baumannii* as a source of global outbreaks and epidemics especially due to its effectiveness in colonizing hospital environments and due to its increasing resistance to commercially available antibiotics, including *β*-lactams, fluoroquinolones, tetracyclines, and aminoglycosides [1–3].* A. baumannii* is associated with hospital-acquired infections which include ventilator-associated pneumonia, bloodstream infections, meningitis, and urinary tract infections (UTI's) [3].

Mechanisms of resistance in* Acinetobacter* strains include efflux pumps, *β*-lactamases, and modifications in porin proteins.* A. baumannii* expresses aminoglycoside-modifying enzymes (AMEs) making them resistant to aminoglycoside antibiotics. Moreover, mutations in the* gyrA* and* parC* genes make them resistant to quinolones [1]. Worthy of mention are the findings of Fournier et al. (2006) who identified AbaR1 resistance islands (86 kb region) encompassing a cluster of resistance genes, namely, ones coding for tetracycline efflux pumps, several AMEs, AmpC, and OXA-10 *β*-lactamases. Genetic analysis of this region also indicated the presence of transposons and genes formerly identified in* Salmonella* spp. and* E. coli *[4].

Enzymatic degradation by *β*-lactamases is the most prevalent mechanism of *β*-lactam resistance in* A. baumannii*. Serine oxacillinases (Ambler class D OXA-type) and metallo-*β*-lactamases (MBLs) (Ambler class B) are *β*-lactamases with carbapenemase activity. OXA-type carbapenemase genes in* Acinetobacter *can be classified into different phylogenetic subgroups: *bla*_OXA-23_, *bla*_OXA-24_, *bla*_OXA-51_, *bla*_OXA-58_, *bla*_OXA-143_, and *bla*_OXA-235_ [5, 6]. The emergence of carbapenem-resistant strains associated with OXA-type *β*-lactamases is increasing as well as their contribution to outbreaks and patient mortality [7]. Another group of recently discovered *β*-lactamases in* Acinetobacter* is the class B *β*-lactamase *bla*_NDM_ and the class A serine carbapenemase *bla*_KPC_ both have a great opportunity to spread to other bacteria due to their location on mobile genetic elements [8].

In addition,* A. baumannii* has other virulence factors that aid in its protection and survival. It has the ability to form biofilms, which are essentially aggregates in which the cells adhere to each other and to a surface in a self-produced matrix of extracellular DNA, polysaccharides and proteins [1]. In* A. baumannii*, outer membrane protein A (ompA) is involved in biofilm formation on abiotic surfaces [9]. Biofilm formation not only helps protect the bacteria against disinfection for instance, but also helps in trading resistance genes between the participating cells.

In this study, we performed in-depth characterization of extremely resistant nonduplicated* A. baumannii* strains isolated from samples of hospitalized patients from all over Palestine except Gaza.

## 2. Materials and Methods

### 2.1. Ethical Considerations

This study was approved by the Medical Research Committee at Caritas Baby Hospital (MRC-014) in Bethlehem, Palestine.

### 2.2. Study Population

Extremely drug resistant* A. baumannii* isolates, all resistant to carbapenems, were collected from 69 patients from Palestinian hospitals all over Palestine with the exception of the Gaza strip. Isolates were collected from five Palestinian districts as follows: Jerusalem (*N* = 2), Bethlehem (*N* = 22), Hebron (*N* = 34), Nablus (*N* = 8), and Ramallah (*N* = 3). The isolates were collected between January 2006 and February 2014 from blood, urine, sputum, and rectal samples.

### 2.3. *A. baumannii* Species Identification

In order to identify* A. baumannii* from the collected* Acinetobacter* spp. isolates, all isolates were identified biochemically and screened for the naturally occurring carbapenemase gene (*bla*_OXA-51_) intrinsic to* A. baumannii *using polymerase chain reaction (PCR) as previously reported [10, 11].

### 2.4. Presence of ISAba1 Element and the Expression of *bla*_OXA-51_ Gene

The isolates were screened for the presence of the IS element, ISAba1, and analyzed for the potential expression of the *bla*_OXA-51_ gene by investigating the presence of the ISAba1 element upstream of the gene [12].

### 2.5. Antibiotic Susceptibility Testing

Antibiotic susceptibility testing (AST) by disk diffusion test was performed for the following antibiotics (ceftazidime 30 *μ*g, cefotaxime 30 *μ*g, ceftriaxone 30 *μ*g, piperacillin-tazobactam 100/10 *μ*g, cefepime 30 *μ*g, sulfamethoxazole 25 *μ*g, amikacin 30 *μ*g, gentamycin 10 *μ*g, ciprofloxacin 5 *μ*g, meropenem 10 *μ*g, and imipenem 10 *μ*g). In addition, the minimal inhibitory concentration of the two antibiotics meropenem and colistin were determined by Etest (BioMérieux, Marcy-l'Étoile, France). AST was performed and interpreted according to the Clinical and Laboratory Standards Institute (CLSI) guidelines [13].

### 2.6. Detection of Antibiotics Resistant Genes and the Biofilm-Producing ompA Gene

Carbapenem- and aminoglycoside-resistant determinants as well as biofilm-producing virulence factors were screened for using standard PCR assay using a PTC-100 Peltier Thermal Cycler (Bio-Rad, USA). The PCR products were then subjected to gel electrophoresis on 1.5% agarose gel for 45 minutes at 85 V and viewed under UV light using the Transilluminator imaging device (Dinco & Rhenium Industries Ltd., Israel). Target genes and their corresponding primers for PCR amplification are mentioned in Table 1. All PCR reactions were done in singleplex with the exception of the OXA-type class D carbapenemases (*bla*_OXA-23_, *bla*_OXA-24_, and *bla*_OXA-58_) which were done in a multiplex PCR reaction as reported in Mostachio et al. (2009). The assays were held in 25 *μ*l volume reactions containing 2x ReddyMix PCR Master Mix with 1.5 mM MgCl_2_ (Thermo Fisher Scientific, USA) with a primer concentration of 20 pmol/*μ*l at standard PCR conditions. A PTC-100 Peltier Thermal Cycler (Bio-Rad, USA) was used for the PCR amplification reactions.

### 2.7. Multilocus Sequence Typing (MLST)

*A. baumannii* bacterial DNA was extracted from 13 representative* A. baumannii* isolates using the High Pure Nucleic Acid Extraction Kit (Roche, Basel, Switzerland). The isolates covered the time period of the study (between 2006 and 2014) such that one isolate was from the year 2006, two were from the year 2007, one was from the year 2008, one was from the year 2010, two were from the year 2011, two were from the year 2012, and four isolates were from the year 2013. PCR reactions were performed for the seven housekeeping genes:* gltA*,* gyrB*,* gdhB*,* recA*,* cpn60*,* gpi,* and* rpoD *as previously reported by Bartual et al. (2005). PCR reactions were performed in 50 *μ*l volume reactions containing 2x ReddyMix PCR Master Mix with 1.5 mM MgCl_2_ (Thermo Fisher Scientific, USA) with a primer concentration of 20 pmol/*μ*l. The PCR conditions were those described by Bartual et al. with an annealing temperature of 55°C for all genes. A PTC-100 Peltier Thermal Cycler (Bio-Rad, USA) was used for the PCR amplification reactions. PCR products were analyzed on a 1.5% agarose gel and visualized using UV light using the Transilluminator imaging device (Dinco & Rhenium Industries Ltd., Israel). Amplified PCR products with the appropriate size were purified using the High Pure PCR Product Purification Kit (Roche) and sequenced using the BigDye® Terminator v3.1 Cycle Sequencing Kit (Life Technology, Carlsbad, USA). The sequences were analyzed and cleaned using Sequencher® software (Gene Codes, MI, USA) and the PubMLST database. Isolates were assigned sequence types (STs) as per the allelic profiles in the* A. baumannii* MLST database (https://pubmlst.org/abaumannii/). The eBURST V3 algorithm (http://eburst.mlst.net/) was used to show phylogenetic relationships among related STs.

## 3. Results

### 3.1. *Acinetobacter* Species Identification

*bla*_OXA-51_ PCR amplification confirmed that all 69 isolates were* A. baumannii*.

### 3.2. Presence of ISAba1 Element and the Expression of *bla*_OXA-51_ Gene

Of the 69 isolates, 68 (99%) carried the ISAba1 element. Of these, only 30 (43.5%) had the IS element upstream of the *bla*_OXA-51_ gene.

### 3.3. *A. baumannii* Susceptibility Testing

**Figure 1:**
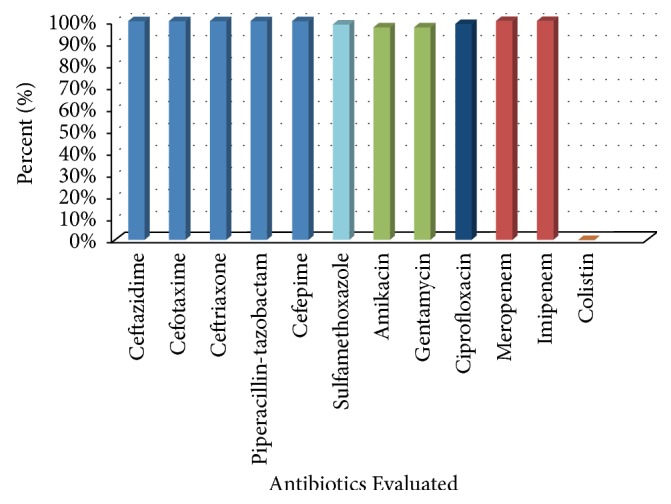
Histogram showing the percent resistance of* Acinetobacter* isolates to each antibiotic tested according to the CLSI guidelines.

All isolates had a multidrug resistant profile, and they were all resistant to all *β*-lactam antibiotics including the carbapenems (Figure 1). The carbapenem resistance profile of all isolates was confirmed by determining the MIC value of meropenem by Etest method (BioMérieux, Marcy-l'Étoile, France). The only class of antibiotics to which the isolates were susceptible was colistin sulfate (100%) (Figure 1). In addition, the majority of* A. baumannii* isolates (>90%) were resistant to the aminoglycosides, macrolides, and cotrimoxazole.

### 3.4. Molecular Characterization

#### 3.4.1. Detection of Carbapenem-Resistant Genes

**Figure 2:**
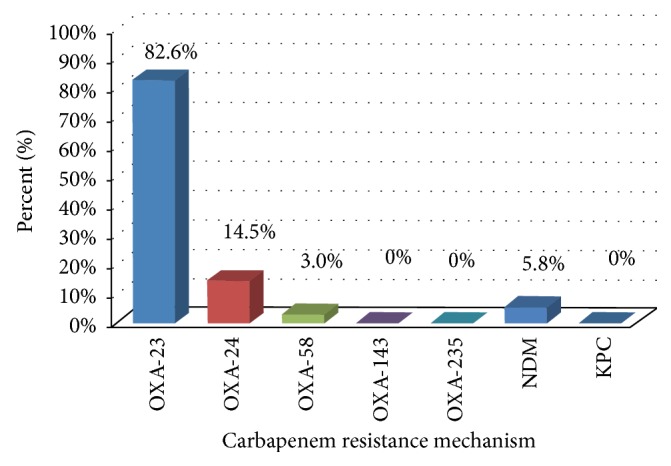
Histogram showing the distribution of carbapenemases in the* Acinetobacter* isolates tested.

Of the 69* A. baumannii* isolates tested, 57 (82.6%) were positive for *bla*_OXA-23_, 10 (14.5%) tested positive for *bla*_OXA-24_, and 2 (3%) tested positive for *bla*_OXA-58_ (Figure 2). None of the isolates were positive for the class D *β*-lactamases *bla*_OXA-143_ and *bla*_OXA-235_. In addition, 3 (5.8%) tested positive for the class B carbapenemase *bla*_NDM_. Sequencing analysis of the *bla*_NDM_ gene revealed that the three isolates carried the *bla*_NDM-1_ gene. None were positive for the class A carbapenemase *bla*_KPC_. It is worthy to note that one MDR* A. baumannii* isolate was a combination OXA-23/OXA-24.

#### 3.4.2. Detection of Aminoglycoside-Resistant Genes

**Figure 3:**
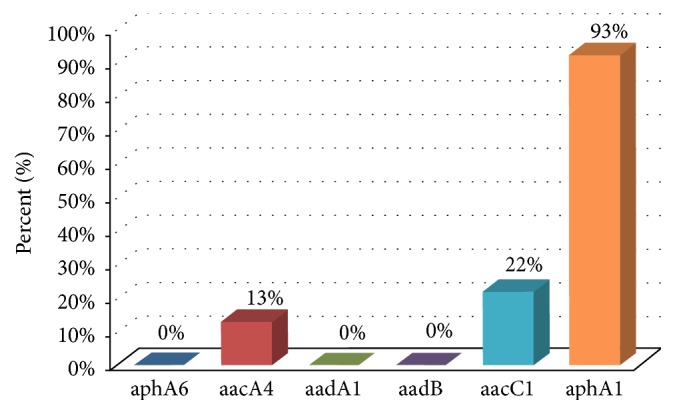
Histogram showing the distribution of AME's genes in the* Acinetobacter* isolates tested.

Of the 69 isolates, 64 (93%) were positive for the* aphA1* gene while none were positive for the* aphA6* gene (Figure 3). On the other hand, the isolates were positive for the acetyltransferases* aacC1 *and* aacA4 *genes in 15 (22%) and 9 (13%) of the isolates, respectively. None were positive for the nucleotidyltransferases* aadB* or* aadA1* genes (Figure 3).

#### 3.4.3. Detection of Biofilm-Producing Virulence Factors

Of the 69* A. baumannii* isolates tested, all (100%) tested positive for* ompA* gene.

#### 3.4.4. Multilocus Sequence Typing (MLST)

**Figure 4:**
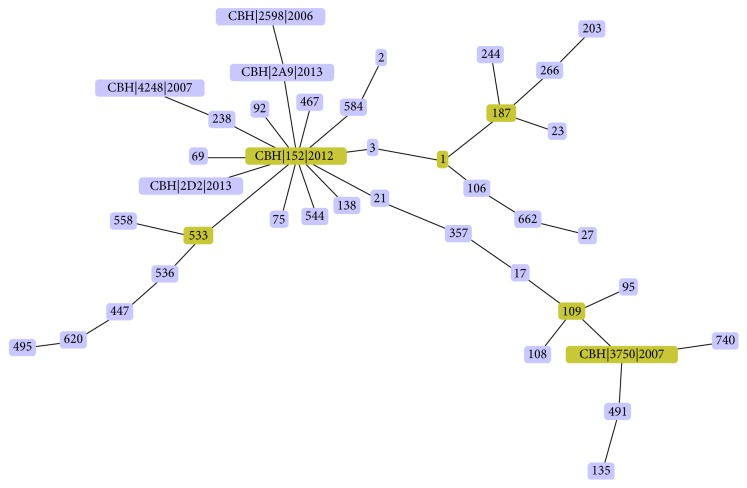
Population snapshot of the 13* A. baumannii* isolates and existing reference isolates obtained from the PubMLST database, represented by the eBURST algorithm (http://eburst.mlst.net/). CBH|152|2012 (ST208) represents the primary founder with a bootstrap value = 1000, where CBH|2598|2006: ST348, CBH|4248|2007: New proposed ST, CBH|3750|2007: ST231, CBH|2A9|2013: ST218, CBH|152|2012: ST208, CBH|2D2|2013: New proposed ST.

Of the 13* A. baumannii *strains evaluated by MLST, the following Sequence Types (STs) were obtained: ST208, ST218, ST231, ST348, and two new Sequence Types suggesting that more than one clone is circulating Palestinian hospitals. Two isolates did not have an exact ST match in the database. The nearest ST match yielded three and four possible STs for the* A. baumannii *isolates CBH|4248|2007 and CBH∖2D2∖2013, respectively. After assigning sequence types for each isolate, phylogenetic relationships among related STs were established using the eBURST V3 algorithm (http://eburst.mlst.net/), and a dendrogram was generated (Figure 4). The central primary founder is ST208 [CBH|152|2012] (bootstrap = 1000). There were 13 linked single locus variants (SLVs) of the founder (ST75, ST544, ST138, ST21, ST3, ST584, ST467, ST218, ST92, ST238, ST69, CBH|2D2|2013, and ST533). Six of the SLVs of the founder (ST533, ST218, ST238, ST584, ST21, and ST3) have diversified to produce double locus variants (DLVs). There were a total of five subgroup founders (ST533, ST1, ST187, ST109, and ST231 [CBH|3750|2007]) that diversified and produced their own SLVs. One of the new STs [CBH|2D2|2013] has only one out of seven of the MLST loci altered when compared to the primary founder genotype, ST208. Its allelic profile has a single locus variant (SLV) from that of the founder's, ST208. On the other hand, the other new ST [CBH|4248|2007] has two out of seven MLST loci altered in comparison to the founder ST208. Hence, its allelic profile has a double locus variant (DLV) from that of the founder's.

## 4. Discussion

*A. baumannii* has acquired a huge genetic repertoire via horizontal gene transfer that makes it virulent and resistant to any environmental pressures [1, 4]. Antibiotic susceptibility testing in this study showed that all* A. baumannii* isolates were resistant to the commercially available antibiotics with the exception of colistin. All isolates had an extremely drug resistant profile and they were resistant to carbapenems. MDR* A. baumannii* outbreaks were reported in hospitals worldwide including the Middle East [14, 15] and this is consistent with the increase in the incidence of health care associated* A. baumannii* infections reported in Palestinian and Israeli hospitals [2, 14, 15].* A. baumannii* isolates from other geographical regions had a similar antimicrobial profile [16]. MIC tests also showed a similar result since most isolates were resistant to meropenem and sensitive to colistin. There are, however,* A. baumannii* isolates that are becoming resistant to colistin [3].

Studying the carbapenem-resistant mechanisms of this pathogen was necessary in order to better control the spread of* A. baumannii* and devise a better antibiotics treatment plan. Carbapenem resistance was mainly attributed to the presence of *bla*_OXA_ genes that produce carbapenem-hydrolyzing enzymes [5]. Since the *bla*_OXA-51_ gene is an oxacillinase naturally occurring mainly in* A. baumannii*, it was present in all* A. baumannii* isolates tested, and the potential expression of this gene in 43.5% of the isolates is facilitated by the IS*Aba1* element present upstream of the gene. The production of *bla*_OXA-23_ by an* A. baumannii *strain is enough to confer resistance to the carbapenems, and 82.6% of the isolates in this study carried this gene. This gene is plasmid-borne suggesting the mobility of this genetic segment facilitating horizontal gene transfer [5]. *bla*_OXA-23_ was also reported in neighboring countries including Iraq, Greece, Italy, and Turkey [16]. Molecular class D *β*-lactamases confer resistance to the carbapenems and narrow-spectrum cephalosporins. 14.5% of the isolates also carried the *bla*_OXA-24_ gene, and 3% of them carried the recently identified *bla*_OXA-58_ gene. This is also the case in neighboring countries as those class D *β*-lactamases have been reported in Iraq, Kuwait, Lebanon, Turkey, Italy, Greece, and the UK [16]. Enzymatic degradation by *β*-lactamases is the most prevalent mechanism of *β*-lactam resistance in* A. baumannii* [7], and this is clearly shown in our sampled* A. baumannii* isolates in the West Bank.

About 6% of our* A. baumannii *isolates tested positive for the class B carbapenemase *bla*_NDM-1_. This is consistent with regional and global results as there are reports in Israel, India, China, United Kingdom, Canada, France, Sweden, Morocco, South Africa, United Arab Emirates, and Iran among many others [8].

The multidrug resistant profile of the isolates was also heightened by the presence of aminoglycoside-resistant determinants, namely, the phosphotransferase* aphA1* and the acetyltransferases* aacC1 *and* aacA4*. Almost all our isolates carried the* aphA1* gene, and 22% and 13% were positive for* aacC1* and* aacA4* genes, respectively. In comparison to other studies, Aliakbarzade et al. (2014) reported the following aminoglycoside-resistant genes in Iran:* aacC1*,* aadA1*,* aphA6,* and* aadB*. Only* aacC1* was detected in our Palestinian isolates, and none of our isolates carried the phosphotransferase* aphA6*. The aminoglycoside-resistant gene* aphA6* was reported in Egypt [17]. On a more international scale, the following aminoglycoside-resistant genes were reported in Poland in 2013:* aphA1*,* aphA6,* and* aacC1* [18]. Our isolates are equipped with not only enzymatic resistance mechanisms, but also the ompA biofilm-producing virulence factor. It is unfortunate that our* A. baumannii* isolates in the West Bank produce biofilms: 100% tested positive for* ompA*.

There is an apparent global outbreak of carbapenem-resistant* A. baumannii* especially in the last decade. MLST is a discriminative typing method whose data are in concordance with other typing results produced by PFGE and AFLP analysis [5]. We sequence typed 13 isolates that are representative samples from different areas and different time periods throughout the West Bank. Sequence analysis of Palestinian genotypes showed a similarity with the global genotypes. Our MLST results reveal that more than one strain of* A. baumannii *is circulating in our Palestinian hospitals; the resulting STs were ST208, ST218, ST231, ST348, and two new STs. We correlated the STs with the timeline of their isolation and preliminary results show that there is a successful spread of clone. To illustrate, ST208 is evident throughout the studied time period, starting in 2008 up till 2014, indicating a successful spread of that particular strain. Internationally, ST208 was reported in the US in 2009, then in Egypt the following year, and then in China in 2012 [19]. Interestingly, the seven ST208 isolates came from the same Palestinian geographical district, Hebron. There are reports of ST218 in the PubMLST database in the year 2000. But, locally in Palestine, our MLST results show that the two isolates, isolated in 2013 from urine and sputum samples, were of ST218. ST231 was first reported in Brazil in 2000 and later again in 2007 in the same country [19]. Our results report ST231 that belongs to a 2007 isolate. Internationally, there are reports of ST348 in the US in 2008 according to the PubMLST database. But this particular ST was detected in Palestine earlier in 2006 as per our MLST results. Worthy of mention is the fact that the isolates carrying the *bla*_NDM-1_ gene did not belong to the same clone.

Moreover, new strains are emerging. According to our MLST results, there is a new strain that emerged in 2007, and another new one in 2013. Over time, the frequent use of antibiotics will result in the emergence of new resistant strains of* A. baumannii *bacteria.

The eBURST V3 algorithm [20] shows the relationship between the closely related isolates in which the founding genotype starts to diversify producing a cluster of closely related genotypes as it increases in frequency in the population. In summary, there is more than one clone of* A. baumannii* circulating worldwide as shown by the sequence analysis of our Palestinian genotypes and the global genotypes.

Resistance to antibiotics, resistance to disinfectants, and resistance to desiccation accompanied by the presence of drug resistant and biofilm-producing genes all contribute to the persistence of* A. baumannii* as a hospital pathogen. Resistant* A. baumannii* strains have a selective advantage in environments like the ICU's where antimicrobials and surface disinfectants are extensively used. Selection for resistant forms can take place during antimicrobial treatment or after treatment. There is a sense of altered microbial ecology with regard to resistant and susceptible bacteria as well as the types of microorganisms surviving after treatment.

Epidemiologically,* A. baumannii* is of concern due to its dissemination mostly in a clonal fashion whether it is within health care institutions and cities or globally between countries. Since* A. baumannii* is easily transmitted and can, therefore, cause outbreaks, certain control measures have to be taken into account. To start with, health care facilities and personnel should follow the CDC's infection control guidelines and protocols, which attempt to control and prevent the transmission of MDR organisms, such as active surveillance, hand hygiene, and contact precautions [19]. Molecular epidemiological studies should be implemented to determine the presence or absence of a clonal outbreak strain such as* A. baumannii *ST208 which appears to be a very successful clone. Proper environmental cleaning of potential reservoir sources present in hospital environments such as ventilators, catheters, containers, and moist articles is highly recommended to prevent the spread of* Acinetobacter.* Moreover, antibiotic stewardship programs must be initiated in order to properly manage treatment and spread of this deadly pathogen.

## Figures and Tables

**Table 1:** Summary of the target genes screened for using polymerase chain reaction (PCR).

Target gene	Primer name	Sequence (5′ to 3′)	Annealing temperature^*∗*^ (°C)	Amplicon size (bp)
Class D carbapenemases	OXA-23	F: GATCGGATTGGAGAACCAGA	58	501
		R: ATTTCTGACCGCATTTCCAT		
	OXA-24	F: GGTTAGTTGGCCCCCTTAAA	58	246
		R: AGTTGAGCGAAAAGGGGATT		
	OXA-58	F: AAGTATTGGGGCTTGTGCTG	58	599
		R: CCCCTCTGCGCTCTACATAC		
	OXA-51	F: TAATGCTTTGATCGGCCTTG	58	353
		R: TGGATTGCACTTCATCTTGG		
	OXA-143	F: TGGCACTTTCAGCAGTTCCT	58	180
		R: TAATCTTGAGGGGGCCAACC		
	OXA-235	F: TTGTTGCCTTTACTTAGTTGC	58	700
		R: CAAAATTTTAAGACGGATCG		

Class B carbapenemases	NDM	F: CCAATATTATGCACCCGGTCG	58	812
		R: ATGCGGGCCGTATGAGTGATTG		

Class A carbapenemases	KPC	F: ATGTCACTGTATCGCCGTCT	58	538
		R: TTTTCAGAGCCTTACTGCCC		

AME's	aphA6	F: ATGGAATTGCCCAATATTATTC	55	797
		R: TCAATTCAATTCATCAAGTTTTA		
	aphA1	F: CAACGGGAAACGTCTTGCTC	58	455
		R: ATTCGTGATTGCGCCTGAG		
	aacA4	F: ATGACTGAGCATGACCTTGCG	65	518
		R: TTAGGCATCACTGCGTGTTCG		
	aadB	F: ATGGACACAACGCAGGTCGC	65	524
		R: TTAGGCCGCATATCGCGACC		
	aadA1	F: ATGAGGGAAGCGGTGATCG	65	254
		R: TTATTTGCCGACTACCTTGGTG		
	aacC1	F: ATGGGCATCATTCGCACATGTAGG	65	456
		R: TTAGGTGGCGGTACTTGGGTC		

Biofilm-producing virulence factors	ompA	F: CGCTTCTGCTGGTGCTGAAT	58	531
		R: CGTGCAGTAGCGTTAGGGTA		
	epsA	F: AGCAAGTGGTTATCCAATCG	58	451
		R: ACCAGACTCACCCATTACAT		

^*∗*^Annealing temperature (°C) used in this study.
